# Supplementary material for: In Vitro Tumor Cell-Binding Assay to Select High-Binding Antibody and Predict Therapy Response for Personalized 64Cu-Intraperitoneal Radioimmunotherapy against Peritoneal Dissemination of Pancreatic Cancer: A Feasibility Study
Source: Int J Mol Sci. 2022 May 22;23(10):5807. doi: 10.3390/ijms23105807 (PMC9146758; doi:10.3390/ijms23105807)
Supplement: Supplementary file 1 [file ijms-23-05807-s001.zip › ijms-1712557-supplementary.pdf]

IJMS

In vitro tumor cell binding assay to select high-binding antibody and predict therapy response for personalized ^{64}Cu-intraperitoneal radioimmunotherapy against peritoneal dissemination of pancreatic cancer: A feasibility study

Running title: In vitro tumor cell binding assay for personalized ^{64}Cu-ipRIT

Fukiko Hihara[1], Hiroki Matsumoto[1,4], Mitsuyoshi Yoshimoto[2], Takashi Masuko[3], Yuichi Endo[3], Chika Igarashi[1], Tomoko Tachibana[1], Mithuhiro Shinada[1,7], Ming-Rong Zhang[1], Gene Kurosawa[4], Aya Sugyo[1], Atsushi B. Tsuji[1], Tatsuya Higashi[1], Kurihara Hiroaki[4], Makoto Ueno[5], Yukie Yoshii[1,4]*

[1] Department of Molecular Imaging and Theranostics, National Institutes for Quantum Science and Technology, Chiba263-8555, Japan; hihara.fukiko@qst.go.jp (H.F.); matsumoto.hiroki2@qst.go.jp (H.M.); igarashi.chika@qst.go.jp (C.I.); tachibana.tomoko@qst.go.jp (T.T.); shinada.mitsuhiro@qst.go.jp (M. S.), zhang.ming-rong@qst.go.jp (M.R.Z.); sugyo.aya@qst.go.jp (A.S.); tsu-ji.atsushi@qst.go.jp (A.B.T.); higashi.tatsuya@qst.go.jp (T.H.)

[2] Division of Functional Imaging, National Cancer Center Hospital East, Chiba277-8577, Japan; miyoshim@ncc.go.jp(Y.M.)

[3] School of Pharmacy, Kindai University, Osaka577-8502, Japan; masuko@phar.kindai.ac.jp (T.M.);en-dou@phar.kindai.ac.jp (Y.E.)

[4]International Center for Cell and Gene Therapy,Fujita Health University,Aichi 470-1192,Japan;gene@fujita-hu.ac.jp(K.G.)

[5]Department of Diagnostic Radiology, Kanagawa Cancer Center, Kanagawa 241-8515, Japan; h-kurihara@kcch.jp(K.H.)

[6] Department of Gastroenterology, Kanagawa Cancer Center, Kanagawa 241-8515, Japan; uenom@kcch.jp(M.U.)

[7] Faculty of Science, Toho University, Chiba 274-8510, Japan**;** shinada.mitsuhiro@qst.go.jp (S.M.)

* Correspondence: yoshii.yukie@qst.go.jp; Tel.: +81-43-206-3426

Correspondence to: Yukie Yoshii, PhD

Department of Molecular Imaging and Theranostics, National Institute of Radiological Sciences, National Institutes for Quantum and Radiological Science and Technology, 4-9-1 Anagawa, Inage, Chiba 263-8555, Japan Tel.: +81-43-206-3426; Fax: +81-43-206-4079; E-mail address: yoshii.yukie@qst.go.jp

Supplemental Data

Figure S1. Western blot images shown in Figure 3.

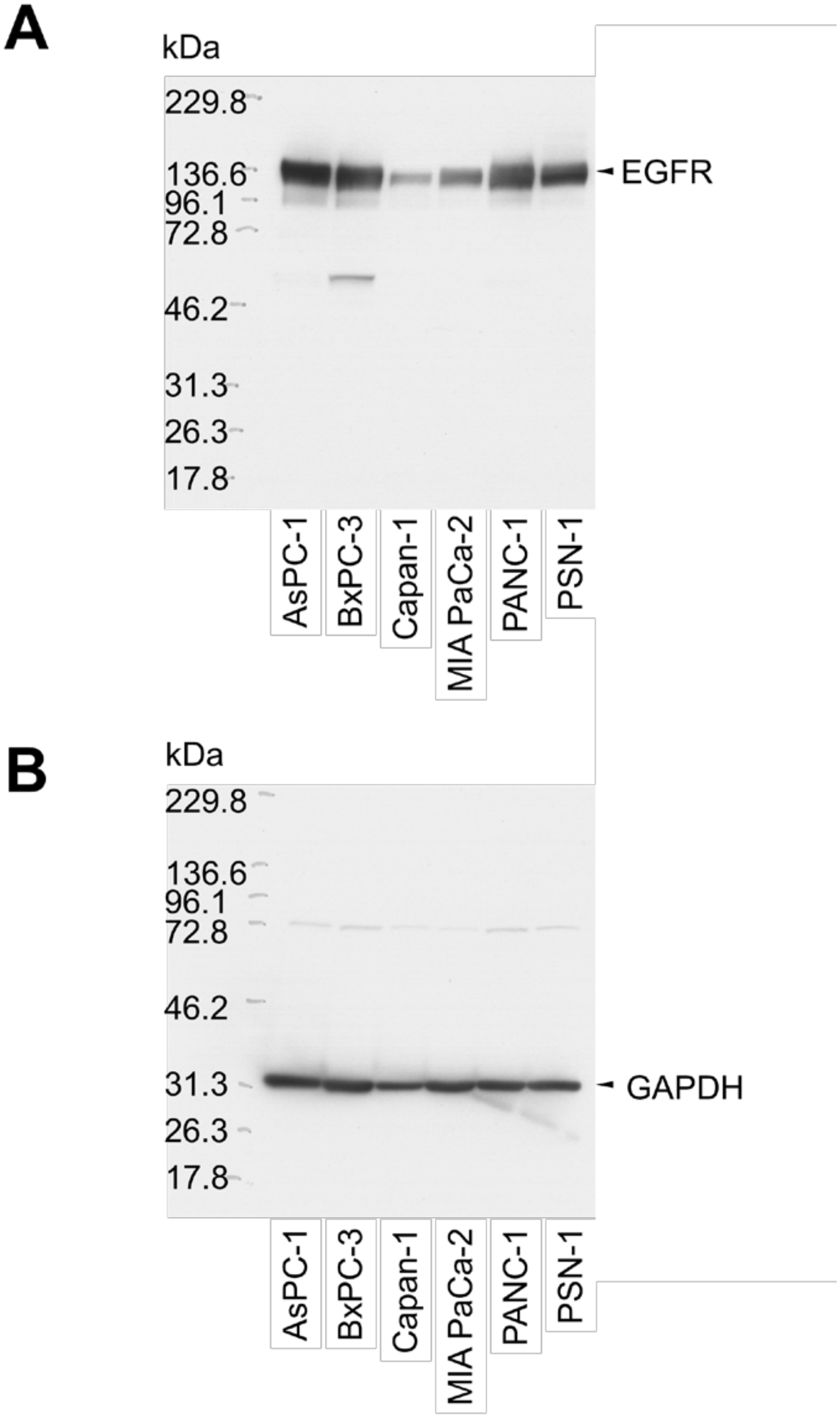

Whole images of western blots for EGFR and GAPDH expression (A and B, respectively).

Figure S2. Changes in body weight during in vivo treatment study.

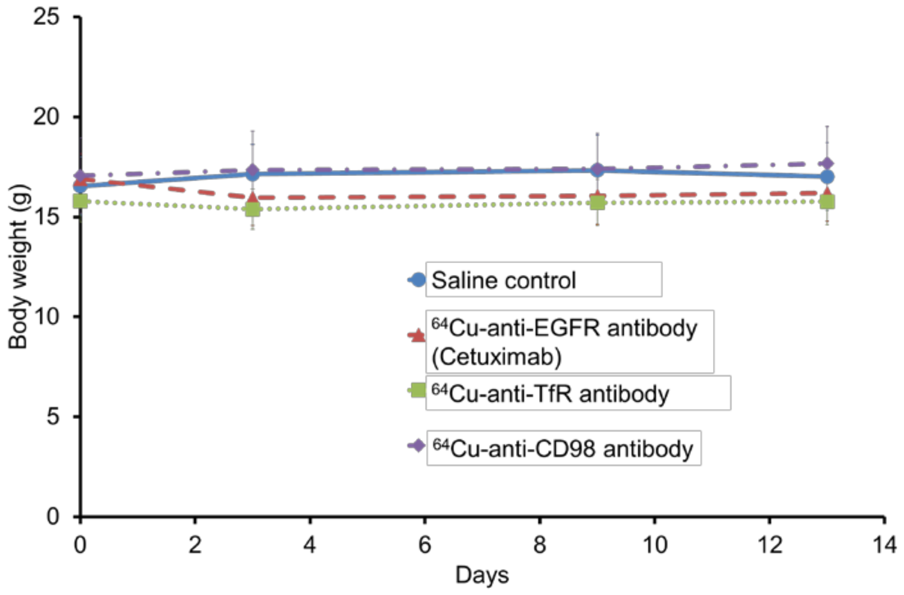

Data from an in vivo ^{64}Cu-ipRIT study using the peritoneal-dissemination models of Capan-1 are shown as a representative example. There was no weight loss of more than 20% compared to the initial body weight in any treatment group for all cell line models.
